# Supplementary material for: Spatial and temporal regularization to estimate COVID-19 reproduction number R(t): Promoting piecewise smoothness via convex optimization
Source: PLoS One. 2020 Aug 20;15(8):e0237901. doi: 10.1371/journal.pone.0237901 (PMC7444593; doi:10.1371/journal.pone.0237901)
Supplement: Attachment — Submitted filename: Responses2Reviewer.pdf [file pone.0237901.s001.pdf]

Laboratoire de Physique de l'École Normale Supérieure de Lyon, UMR CNRS 5672
46, allée d'Italie, F-69364 LYON CEDEX 07, FRANCE
Tel. : (+33) 4 72 72 81 42
Email: patrice.abry@ens-lyon.fr

July 31, 2020

Dear Reviewer,

We would like first to thank you a lot for the work and time dedicated to the review of the manuscript entitled "Spatial and temporal regularization to estimate COVID-19 Reproduction Number $R(t)$: Promoting piecewise smoothness via convex optimization", by my research group and myself, submitted to PlosOne Journal as a research Article.

We have worked on the revised version as requested. Please find below detailed responses to comments, requests and questions either from the editorial board or from the reviewer. The corrections are marked by colors: text in blue indicates what has been suppressed from original submission and text in red stands for additions or replacements.

We thank the reviewer for the overall appreciation, detailed comments and relevant suggestions. We provide below detailed responses to the different points raised by the reviewer.

Context: To enrich the work of interest it might be useful to explain the practical usefulness of having new predictive models inserting some sentences such as:??The ability to provide a predictive model is extremely useful for management of patients and also for medical resource allocation. What was in fact achieved in recent months of February and March was the difficulty to have models that could predict the number of patients (not only the infected) and what was the real need for personal protection and support for patients in hospitals.?At that time, in fact, the data and methods of spreading the epidemic in China were not fully available, but already in Europe, primarily in Italy, the epidemic was spreading. In fact, we were not talking about a pandemic yet and we were not able to meet the needs of gloves, disinfectant, facial mask, mechanical ventilators and beds of intensive care?. The goal of the entire research community is to find a solution to provide timely data to prevent future health crises.?To support these sentence authors can cite these COVID-19 update articles or others that they can choose:

Coccia M. Factors determining the diffusion of COVID-19 and suggested strategy to prevent future accelerated viral infectivity similar to COVID.?Sci Total Environ. 2020 Aug 10;729:138474. doi: 10.1016/j.scitotenv.2020.138474.

?Petkova E, Antman EM, Troxel AB.?Pooling Data From Individual Clinical Trials in the COVID-19 Era?JAMA. Published online July 22, 2020. doi:10.1001/jama.2020.13042

?Ing, RJ; Bills, C; Merritt, G et al.?Role of Helmet-Delivered Noninvasive Pressure Support Ventilation in COVID-19 Patients.?J Cardiothorac Vasc Anesth; 2020 May 08. doi: 10.1053/j.jvca.2020.04.060.?

Agreed, we have amended the first part of the introduction to take into account these relevant suggestions. Also, we quoted the two first suggested papers.

The part ?issue and related work? is too long: it is recommended to eliminate the details that will be described later to

better focus the work points.

Agreed and done. We have removed Equation and Model form the Introduction and develop it only in Section 2.

MATERIALS AND METHODS : To make the reading clearer it is necessary to delete parts already described or comments and just describe the data.?Model and estimation procedures represent the core of the work and unnecessary or repeated parts should be eliminated (for example line 125-127 ?to enable..basis?or line 151-152 ?outliers?ways? or line 167-169 ?as?outbreak?).

Agreed and done. We have further removed some useless repetitions. Also, we have corrected a typo in the Equation after Equation 10.

RESULTS : The study presents the results of original research.?Results reported have not been published elsewhere.?Methods are described in sufficient detail for another researcher to reproduce the experiments described.

We thank the reviewer for this appreciation, we are indeed aiming toward open and reproducible science.

CONCLUSIONS : In order to clarify the conclusions it is necessary to delete some phrases or concepts already described (for example line 520-532 ?Estimation?.analysis?; line 546-552 ?As previously? address?).?I suggest to move the sentence ?when decisions need to be taken and action need to be made 525?under emergency? (line 525-526) at the end of the conclusion paragraph (line 566).

We agree that the conclusion section was somehow hard to follow as it was mixing discussions and future works. We have reorganized that section to enhance clarity.

Best regards,

Patrice Abry
